# New-onset axillary lymphangioma: a case report

**DOI:** 10.1186/s13256-022-03461-0

**Published:** 2022-06-19

**Authors:** Sasha Kurumety, Michael Morris, Zeynep Bostanci Aydi

**Affiliations:** 1 Department of Internal Medicine, University of Arizona College of Medicine-Phoenix, 1111 East McDowell Rd., Phoenix, AZ 85006 USA; 2 Department of Radiology, Banner University Medical Center-Phoenix, 1111 East McDowell Rd., Phoenix, AZ 85006 USA; 3 Department of Surgery, Banner MD Anderson Cancer Center-Phoenix, University of Arizona College of Medicine-Phoenix, 925 East McDowell Rd., Phoenix, AZ 85006 USA

**Keywords:** Axillary lymphangioma, Adult lymphangioma, Cystic hygroma, Case report

## Abstract

**Background:**

Lymphangioma is a rare diagnosis in adult patients and typically presents in early infancy. These tumors are a result of malformation of the lymphatic vessels and usually involve the head, neck, and axilla.

**Case presentation:**

We report the case of a 28-year-old African female who recently immigrated from East Africa and presented to our surgical breast clinic with a large and rapidly growing left axillary mass. Initial history and evaluation were concerning for hydatid cyst; however, on surgical excision, gross appearance was consistent with cystic lymphangioma. Diagnosis was confirmed on pathology review.

**Conclusions:**

Although lymphangiomas are typically found in young children, adults may develop these tumors in response to unknown triggers. Surgical excision is the preferred treatment.

## Background

Lymphangiomas are uncommon benign congenital malformations of the lymphatic system that typically present in childhood. Of lymphangiomas, 50% are present at birth, with 90% of lymphangiomas presenting by 2 years of age [1]. These tumors mostly occur in the head and neck region (75%) or the axilla (20%) [2]. Though they are benign, these tumors can grow quite large and become disfiguring. Depending on the location, they can even impair normal swallowing/breathing function, or in the case of axillary lymphangiomas impair normal range of motion of the arm. Given their congenital nature, lymphangiomas rarely present in adulthood, and very few cases of adult axillary lymphangioma have been reported in literature [2–7].

Meanwhile, hydatidosis and infections with *Echinococcus granulosus* occur worldwide, with prevalence of up to 10% in hyperendemic areas of East Africa [8, 9]. Hydatid cysts most often form within the liver in human hosts but may develop anywhere throughout the body. Here, we describe an unusual case of a large cystic axillary lesion in an adult female with concern for both lymphangioma and hydatid cyst on initial workup. While imaging was suggestive of lymphangioma, the diagnosis was not confirmed until surgical intervention.

## Case presentation

A 28-year-old African female with history of three prior pregnancies with full-term deliveries and recent immigration from East Africa presented with a large, tender left axillary mass. The patient noted the initial appearance of the mass 3 years prior, at which time, the mass was small and nontender. The initial appearance of the mass was 6 months after having an etonogestrel implant device placed within the left arm for contraception, 8 months after her most recent pregnancy and delivery, and 2 years after immigrating to the USA from East Africa. The mass continued to grow slowly for 2.5 years without any pain, drainage, overlying skin changes, or changes in sensation or strength of the left arm. The patient did not experience any difficulty with breastfeeding. Six months prior to presentation, the patient noticed rapid growth and increasing tenderness of the axillary mass that prevented full arm adduction. The patient reported having her etonogestrel implant device removed and replaced 2 months prior to the onset of rapid growth. She denied any breast tenderness, abnormal discharge, or arm weakness. The patient also denied any preceding trauma or infection in the area. The patient had no other significant past medical history or family history, and no recent environmental or employment-related exposures. Social history was significant only for immigration from East Africa 5 years prior to presentation. The patient had no alcohol, tobacco, or illicit substance use and took no regular medications other than the etonogestrel implant device. On initial presentation to our clinic, physical examination demonstrated a protruding, nonreducible, mobile, softball-sized mass in the left axillary fossa with overlying skin striations. The patient did not have any cervical or clavicular lymphadenopathy. She had no vascular or neurological deficits of the left arm, and both sensory and motor function were intact. Due to the size of the mass, the patient was unable to fully adduct the left arm and had mild discomfort with adduction. Vital signs, physical examination, and neurological examination were otherwise unremarkable.

Prior to presentation to our clinic, the patient had undergone magnetic resonance imaging (MRI) with and without contrast, which demonstrated a 16 × 11 × 11 cm^3^ multiloculated subcutaneous cystic lesion in the left axilla. There was displacement but no invasion of surrounding structures, no intrathoracic communication, and no axillary lymphadenopathy. Based on this imaging, lymphangioma was suspected, but hydatid cyst could not be ruled out given the patient’s history of immigration from an endemic location. To further classify the lymphatic and vascular involvement of the mass, MR lymphangiogram was obtained by injecting contrast media subcutaneously into each interdigital web space of the left hand.

**Fig. 1:**
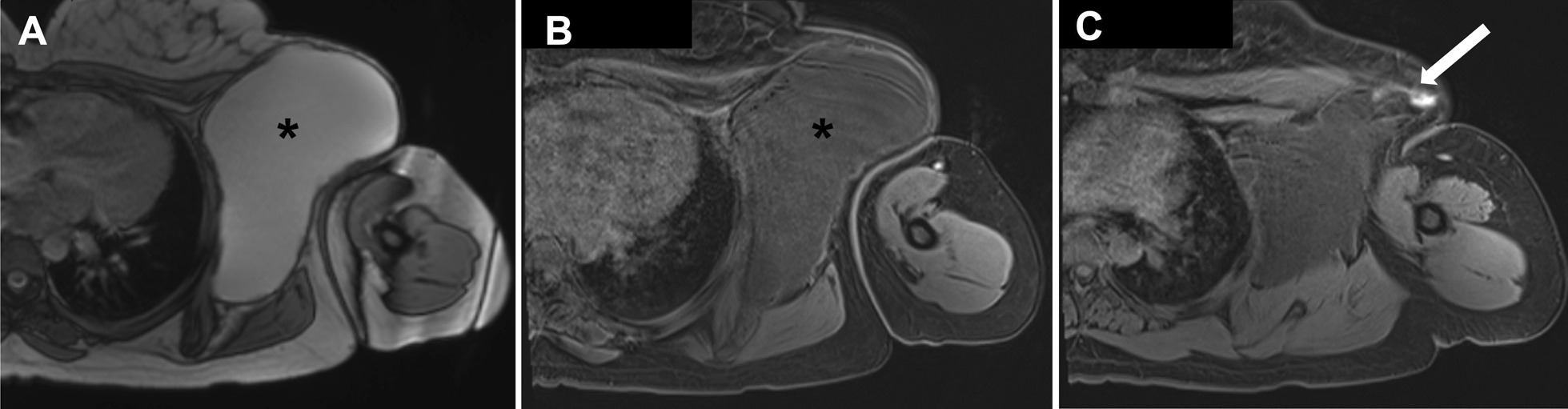
Axial images from MRI lymphangiogram. **A** Noncontrast steady-state free precession demonstrating large cystic mass centered in the left axilla (*). **B** Ten-minute post contrast image following subcutaneous injection of contrast into the web space of the hand, demonstrating no enhancement of the mass (*). **C** Post-contrast image demonstrating enhancement of an axillary lymph node overlying the mass (arrow)

Lymphangiogram showed no evidence of active lymphatic communication with the mass. Lymphatics in the left upper extremity drained to nodes overlying the large cystic mass (Fig. 1). Additionally, echinococcal IgG antibody was tested, with equivocal results. Initial laboratory results did not suggest systemic infection and were otherwise unremarkable. Preoperative pharmacotherapy for *Echinococcus* was considered but deferred since the diagnosis was not confirmed.

**Fig. 2:**
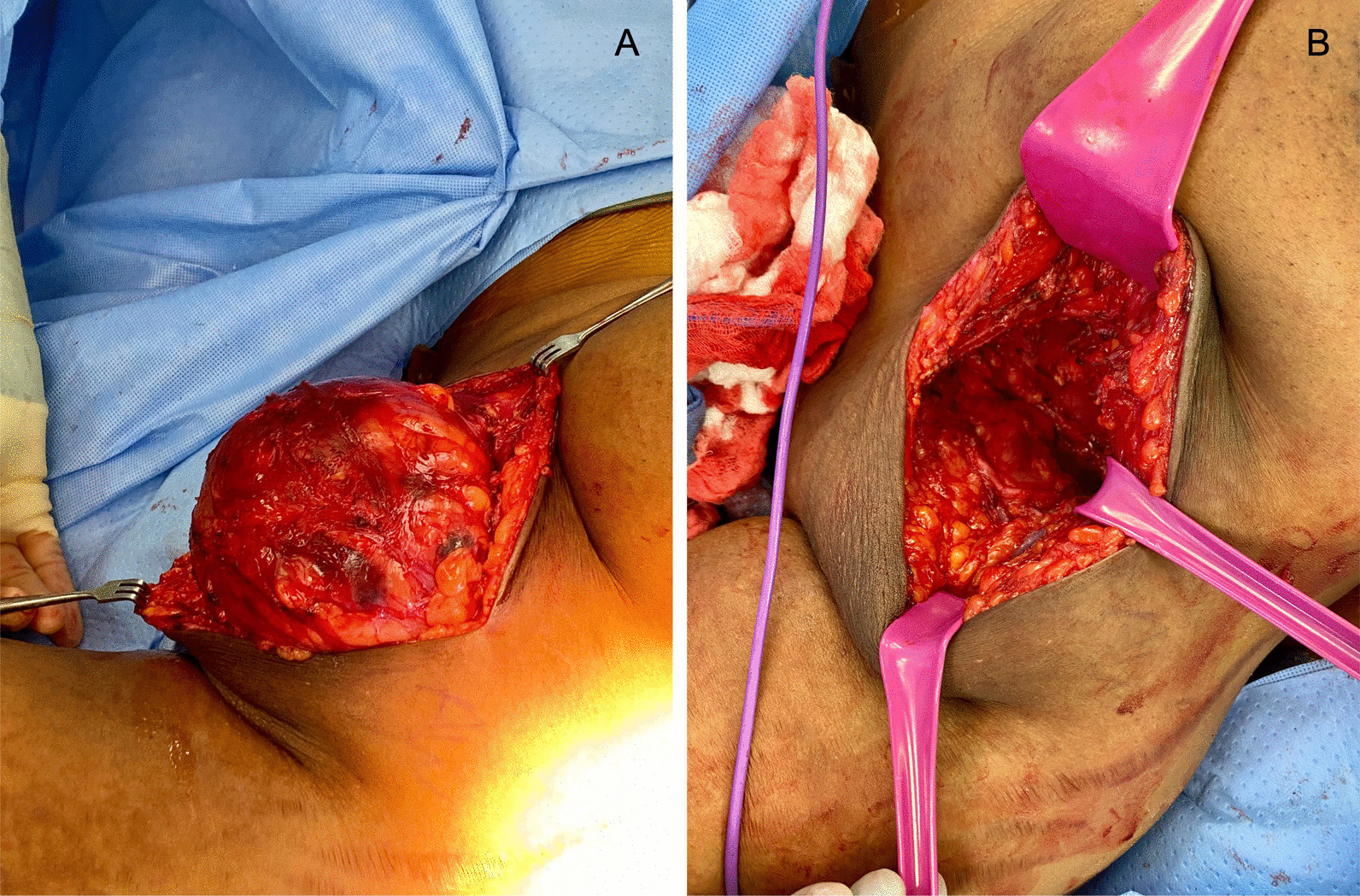
Intraoperative images of mass excision. **A** Exposed mass within the left axilla. **B** Post-excision pocket after mass removal

After review of the images and laboratory results, treatment options including percutaneous drainage and surgical excision were discussed with the patient. Given the large size of the mass with presence of overlying stretched skin, surgical excision was recommended. The patient consented and underwent complete surgical excision of the mass along with the overlying skin. Given the possibility that the mass was a hydatid cyst, the surgical field was prepared with sterile 20% hypertonic saline-soaked gauze as a protoscolicidal agent, as reentry of immature tapeworms into the patient would cause anaphylaxis. Intraoperatively the mass was found to be translucent with very thin walls, and drainage demonstrated typical yellow cystic fluid. The appearance of the cyst and fluid on direct visualization ruled out hydatid cyst, which would appear with thicker, opaque white walls. Therefore, cultures of the cystic fluid were not sent. After excision of the mass there was a large pocket within the axillary tissue, and a Jackson–Pratt drain was placed to prevent seroma development (Fig. 2).

**Fig. 3:**
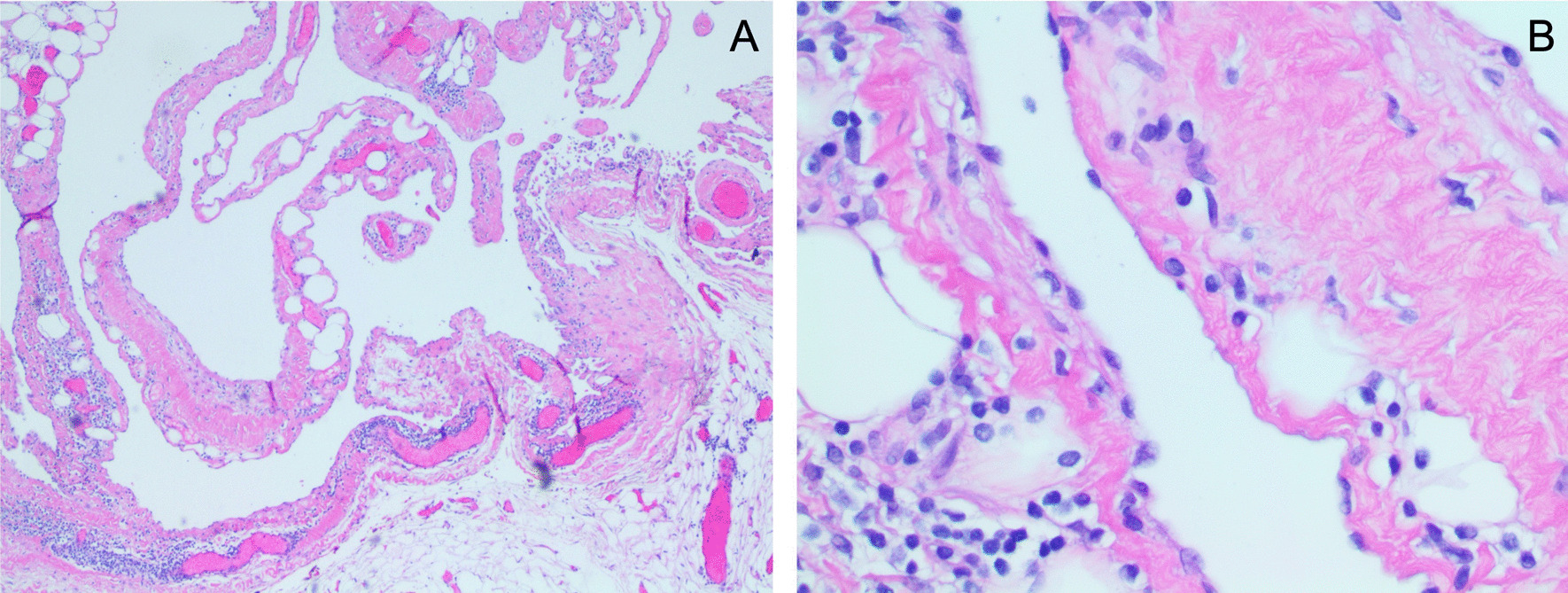
Pathology review of surgical specimen consistent with lymphangioma. **A** Thin-walled vascular channels lacking blood within the lumens. **B** Lymphoid aggregates within tissues

Gross surgical pathology showed a collapsed cyst measuring 9.5 × 9.0 × 4.0 cm^3^. Microscopic evaluation of the specimen showed large lymphatic spaces lined by thin epithelium, morphologically most consistent with cystic lymphangioma (Fig. 3). Associated lymph nodes were evaluated and determined to be histologically benign.

Approximately 1 month after surgery, the patient developed a surgical-site infection, which presented as a change in the quality of the JP drain output from serous to purulent. Drain fluid cultures were collected with resulting growth of *Pseudomonas aeruginosa* and methicillin-sensitive *Staphylococcus aureus* (MSSA). Blood cultures collected at this time were negative. Based on drain culture susceptibilities, the patient was started on standard dosing of oral amoxicillin/clavulanate and ciprofloxacin, with rapid resolution of infection. The drain was subsequently removed. At 6 month follow-up, the patient does not have any re-accumulation of fluid or signs of infection.

## Discussion and conclusions

Adult presentation of lymphangioma is extremely rare. Therefore, in our patient who had recently immigrated from a hyperendemic area of East Africa, an echinococcal hydatid cyst was high on the differential for this rapidly growing axillary cystic mass. While literature reports of adult-onset lymphangioma are already limited, this case is additionally unique as the surgical team had to prepare for a potentially life-threatening intraoperative anaphylactic reaction if direct visualization confirmed the mass to be a hydatid cyst rather than a benign lymphangioma.

Initial differential diagnoses for this case also included simple cyst, abscess, and cavernous hemangioma. To differentiate these diagnoses, we obtained MRI imaging as well as *Echinococcus* IgG antibody testing. MRI demonstrated a large multiloculated cyst in the left axilla with low T1 and high T2 intensity, an appearance most consistent with lymphangioma. Imaging was not consistent with typical appearance of abscess, simple cyst, or hydatid cyst. Typical active hydatid cysts have a complex appearance with daughter vesicles and internal layering due to the hydatid sand that forms with rupture of vesicles [10]. Additionally, hydatid cysts are most often found within the liver. Though this is the common radiographic appearance, characteristics of the cyst may differ in an active versus inactive stage, and hydatid cysts can occasionally be found in extrahepatic locations [11]. IgG testing was performed with equivocal results, indicating possible but not definitive presence of *Echinococcus*. Based on the combination of imaging and equivocal IgG results, hydatid cyst could not definitively be ruled out.

Intraoperative rupture of a hydatid cyst was a significant concern during excision, as hydatid fluid is highly toxic and direct exposure can cause a fatal anaphylactic reaction [12]. Therefore, the operative field was arranged to prevent skin or tissue contact with cystic fluid should rupture occur. However, on direct visualization of the cyst, it appeared deformable, translucent with pale-yellow cystic fluid, and without daughter cysts. In contrast, a hydatid cyst would appear round or ovoid with opaque walls and within a fibrous pericyst formed from host tissue. The diagnosis of macrocystic lymphangioma was confirmed on final pathology.

The exact etiology of adult lymphangioma is not clear. However, it has been proposed that adult presentation and growth of lymphangiomas may be related to a predisposing trigger, such as trauma, infection, or iatrogenic. In older children, it is also thought that puberty can serve as a trigger for growth of lymphangiomas, possibly because of rapid physiological and hormonal changes [1]. Given the hormonal and physiological changes that occur in pregnancy, this may also serve as a trigger. In our patient, presentation of the lymphangioma coincided with her pregnancy as well as placement of a Nexplanon device, which may have served as a hormonal trigger as well.

Lymphatic malformations can be classified based on their radiographic features into three groups: macrocystic, microcystic, and mixed. Differentiation is based on the size of the fluid-containing tissues within the malformation. The macrocystic type (also known as cystic hygroma) is made up of large cysts > 2 cm in diameter, while the microcystic type (lymphangioma circumscriptum) is composed of cysts < 2 cm in diameter [1]. Our patient was confirmed to have a macrocystic lymphatic malformation based on the size of cystic spaces on pathology. All three subtypes are managed similarly, with complete surgical excision providing the lowest rate of recurrence [13, 14]. Alternatively, percutaneous aspiration with injection of sclerosing agents (sclerotherapy) may be considered depending on the size, location, and clinical presentation [15]. Ultimately, the trigger for lymphangioma growth in our patient is unknown, but annual imaging and surveillance is not indicated as long as the patient remains asymptomatic.

## Data Availability

Data reported on in this study are not publicly available as they were directly obtained from the patient’s electronic medical record. All publicly available cited works can be found in the References section.
